# Cleavage Intercondylar Fracture of the Distal Femur: A Report of Two Cases From Uttarakhand, India

**DOI:** 10.7759/cureus.72535

**Published:** 2024-10-28

**Authors:** Ganesh S Dharmshaktu, Ajay Kamat, Krishna Dev S Yadav

**Affiliations:** 1 Department of Orthopaedics, Government Medical College, Haldwani, IND; 2 Department of Orthopaedics, Sindhudurg Shikshan Prasarak Mandal (SSPM) Medical College and Lifetime Hospital, Sindhudurg, IND

**Keywords:** cleavage fracture, distal femur fractures, intercondylar fracture, intra-articular fractures, traumatic knee injury, undisplaced fracture

## Abstract

Intraarticular knee fractures are important injuries and require optimal management for good outcomes. Cleavage fractures are rare variants that present with an undisplaced fracture involving a small area in the intercondylar region of the distal femur. These fractures neither propagate into the metaphysis nor involve any distal femoral condyle (medial or lateral). Their occurrence is limited to a few sporadic reports, and not much is available in the medical literature. We hereby report our experience with two cases, one of a young adult and one of an adolescent. Most of these fractures are successfully managed by non-operative methods, just as in our adult patient. The adolescent patient was managed by minimal-invasive cannulated screw fixation along with the ipsilateral proximal femoral fixation. Excellent healing and full return of function were reported in both cases, in follow-ups of nine and eleven months, respectively.

## Introduction

Intercondylar cleavage fractures are rare injuries and there are sporadic reports in the medical literature. The first description, with a series of three cases, was reported by Pogrund et al. in 1981 [1]. All three cases were managed conservatively with good outcomes. Injury to the intercondylar femur with a force transmitting through the patella results in fractures in this peculiar region [1,2]. In these injuries, a vertical fracture line is noted in the intercondylar area without separating the condyles [2]. These injuries are also not specifically described in widely used classifications of distal femur fractures [3]. More acknowledgment and recognition of this rare injury is required for proper reporting and comprehensive knowledge.

## Case presentation

Case 1

**Figure 1:**
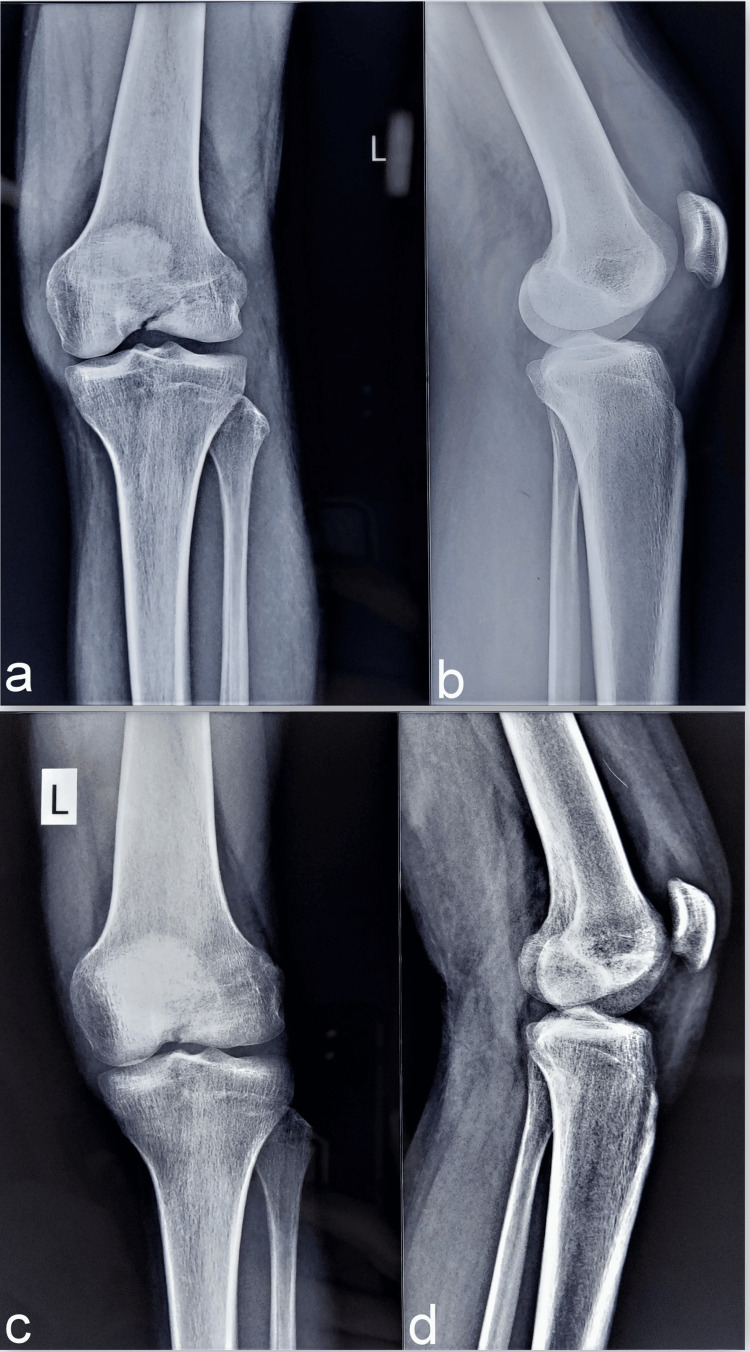
The knee orthogonal radiographs (a, b) show the undisplaced and incomplete fracture in the intercondylar region of the knee. The fracture is not traversing the distal femoral condyles. The radiographs at the final follow-up (c, d) show the healing of the fracture following conservative management.

A 22-year-old male patient presented to the emergency department following a fall on the ground from a height of 3 meters. The fall caused a right knee injury along with acute pain and difficulty in weight bearing on the affected extremity. There was no other injury, and distal neurovascular status was intact. Knee radiographs were done and revealed an incomplete, non-displaced fracture contained in the intercondylar region (Figures 1a, 1b).

The fracture neither had any propagation into the distal femur condyles nor metaphyseal extension. This rare injury pattern is described as a ‘cleavage intercondylar fracture’ of the distal femur. The conservative management with a long knee brace, pain medication, and quadriceps strengthening exercises for six weeks led to the gradual healing of the fracture. Full regain of movement was noted along with sound radiological healing at the final follow-up of nine months (Figure 1c, 1d). No clinical evidence of knee instability was noted as he performed activities of daily living without pain at the final follow-up.

Case 2

**Figure 2:**
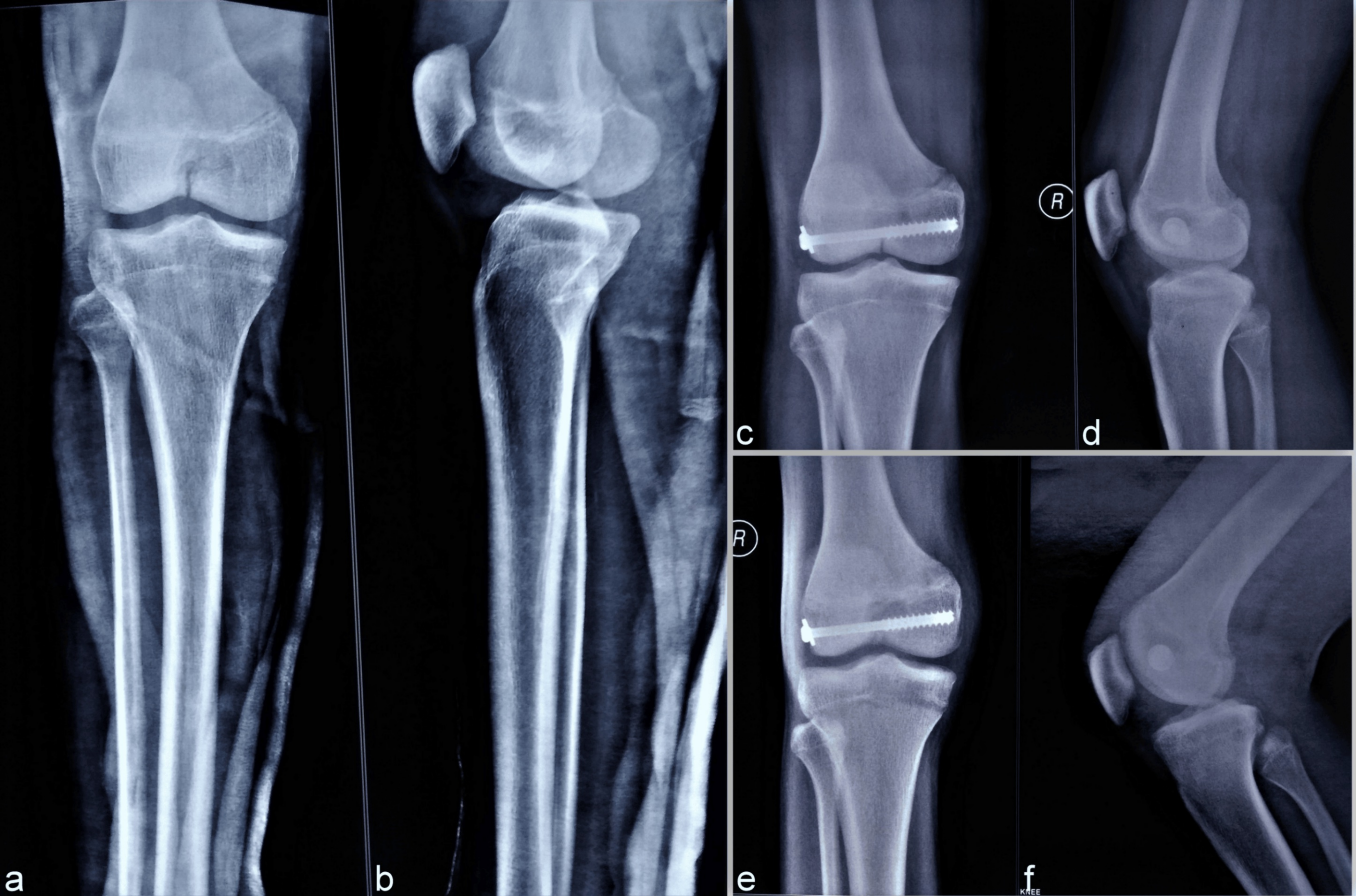
Radiographs show the presence of an undisplaced, incomplete fracture noted in the intercondylar distal femur area, both in the anteroposterior (a) and lateral (b) views. The fracture is not extending into either condyle. The fracture was fixed with a percutaneous screw fixation and showed good placement in anterioposterior (c) and lateral (d) views. Good union was achieved in the follow-up at 11 months in orthogonal (e, f) views with stable knee and full range of motion.

A 17-year-old male child, presented with a history of a road-traffic accident resulting in simultaneous left-sided knee and hip injury with acute pain, deformity, and disability in weight bearing. He was rushed to the emergency room and given the first aid. There was no open injury and tenderness over the left hip and knee region was present. Distal neurovascular status was intact and the pelvic radiograph revealed left side intertrochanteric fracture. A knee radiograph revealed an incomplete, minimal displaced fracture in the intercondylar distal femur area. The fracture did not propagate into any femoral condyles (Figures 2a, 2b). The distal femoral physeal line, however, was also appreciated in the radiograph but was distinct from the aforementioned fracture. As the intertrochanteric fracture required fixation, the parent consented to simultaneous fixation of cleavage fracture for earlier rehabilitation. Another reason for making this choice was that he was an amateur soccer enthusiast.

The intertrochanteric fracture was fixed with a cephalomedullary nail and a percutaneous cannulated cancellous screw was used to provide stability to the cleavage fracture (Figures 2c, 2d). The screw was aimed to provide restraint to inadvertent fracture propagation, though unlikely, and earlier rehabilitation. Gradual healing of the cleavage fracture was noted in the coming four months and at the final follow-up at 11 months, the fracture was fully united (Figures 2e, 2f) without clinical knee instability.

## Discussion

Distal femur fractures have bimodal age distribution with traumatic etiology in younger patients and trivial osteoporosis-related injuries in the older age group [4]. These fractures are also classified into extra-articular, partial articular, and complete articular types in widely used classification by the Arbeitsgemeinschaft für Osteosynthesefragen/Orthopedic Trauma Association (AO/OTA) system [3,4]. Cleavage fractures, incomplete and undisplaced fractures limited to the intercondylar region, are not described as distinct entities. These are rare injuries with only a handful of cases reported so far in the medical literature [1,2].

Forces acting through the patella with a direct force transmission through a 60-90 degree flexed knee, are causative factors for the cleavage fractures [1,3,4]. These, however, may be considered intercondylar fractures with no separation of the femoral condyles. As these injuries are linear and undisplaced, they can be managed conservatively with good outcomes. A long leg splint or cast for six to eight weeks, with periodic monitoring for fracture displacement, is usually sufficient for a good outcome.

Anatomical articular reduction is stated to be ideal, but the decision is also based on individual characteristics, patient demands, and activity level [5]. The first case was managed conservatively by us, and the second was operated by a minimally invasive method for earlier rehabilitation as he demanded an earlier return to amateur sports activity. Bone quality, absence of co-morbidities, and fracture characteristics are important parameters affecting overall outcomes in distal femur fractures [4,5]. Both the patients reported here, being otherwise healthy and young, had excellent outcomes based on positive characteristics in this regard.

One shortcoming, however, in our report is the absence of advanced imaging, like CT or MRI scans, that would have given additional information regarding associated injuries. Associated knee and ligamentous injuries are described in good frequency in the settings of femoral shaft fracture [6]. The chance of associated meniscal or ligamentous injuries with cleave fractures, therefore, can not be ruled out. Clinically, however, no significant knee effusion or pain was noted. The above investigations were withheld primarily due to clearly visible uncomplicated fracture patterns and secondarily due to financial constraints. Cleavage fracture is an uncommon entity and more scientific data is required for their comprehensive knowledge. This report, hopefully, shall enrich the literature and be of educational value for students and practitioners alike.

## Conclusions

Cleavage intercondylar distal femur fractures are rare fractures and have not been described much in the literature. Our report describing two cases (one managed conservatively and the other with a minimally invasive screw fixation method) with successful healing and good functional outcome may enrich the medical literature. These fractures can be managed by both the aforementioned methods with good outcomes. The fixation of these fractures has not been reported in any prior work as per our knowledge and the option may be limited to selected cases, in our opinion. We believe that many of these cases are missed, casually overlooked, and thus underreported in the practice, and better identification and reporting shall promote more studies on their biomechanics, associated injuries, and treatment consensus. Robust studies in the future shall improve our understanding of this peculiar injury pattern. 
